# DUIncoder: Learning to Detect Driving Under the Influence Behaviors from Various Normal Driving Data

**DOI:** 10.3390/s25061699

**Published:** 2025-03-09

**Authors:** Haoran Zhou, Alexander Carballo, Masaki Yamaoka, Minori Yamataka, Keisuke Fujii, Kazuya Takeda

**Affiliations:** 1 Graduate School of Informatics, Nagoya University, Furo-cho, Chikusa-ku, Nagoya 464-8603, Japan; fujii@i.nagoya-u.ac.jp (K.F.); kazuya.takeda@nagoya-u.jp (K.T.); 2 Graduate School of Engineering, Gifu University, 1-1 Yanagido, Gifu City 501-1193, Japan; alex@gifu-u.ac.jp; 3 Institute of Innovation for Future Society, Nagoya University, Furo-cho, Chikusa-ku, Nagoya 464-8601, Japan; 4 Tier IV Inc., Nagoya University Open Innovation Center, 1-3, Mei-eki 1-chome, Nakamura-ku, Nagoya 450-6610, Japan; 5 HMI R&I Department, Advanced Research and Innovation Center (ARIC), DENSO CORP., 500-1, Minamiyama, Komenoki-cho, Nisshin 470-0111, Japan; masaki.yamaoka.j4j@jp.denso.com (M.Y.); minori.yamataka.j6y@jp.denso.com (M.Y.); 6 Institute of Physical and Chemical Research (RIKEN) Center for Advanced Intelligence Project, 1-5, Yamadaoka, Suita 565-0871, Japan

**Keywords:** driving behavior, driving under influence, unsupervised learning, increment learning

## Abstract

Driving Under the Influence (DUI) has emerged as a significant threat to public safety in recent years. Despite substantial efforts to effectively detect DUI, the inherent risks associated with acquiring DUI-related data pose challenges in meeting the data requirements for training. To address this issue, we propose DUIncoder, which is an unsupervised framework designed to learn exclusively from normal driving data across diverse scenarios to detect DUI behaviors and provide explanatory insights. DUIncoder aims to address the challenge of collecting DUI data by leveraging diverse normal driving data, which can be readily and continuously obtained from daily driving. Experiments on simulator data show that DUIncoder achieves detection performance superior to that of supervised learning methods which require additional DUI data. Moreover, its generalization capabilities and adaptability to incremental data demonstrate its potential for enhanced real-world applicability.

## 1. Introduction

According to the 2021 statistics from the World Health Organization (WHO) [1], road traffic accidents resulted in 1.19 million fatalities globally, amounting to 15 deaths per 100,000 individuals, ranking among the foremost causes of death across all age groups. It also indicates that 20% to 69% of the drivers involved across different countries were Driving Under the Influence (DUI) of alcohol or drugs with excessive alcohol consumption being a notable factor. To improve traffic safety, many countries have undertaken measures to reduce drunk driving. For example [2], since the 1970s, Japan has implemented legislative reforms, conducted public awareness campaigns, and intensified the enforcement of drunk driving laws. However, the effectiveness of these efforts appears to have stagnated in recent years. Between 2007 and 2017, there has been an increase in the proportion of accidents and fatalities related to drunk driving; notably, the percentage of fatal accidents due to drunk driving rose from 4.9% in 2015 to 5.5% in 2017. Adopting new strategies to reduce drunk driving has recently become particularly imperative.

Currently, the most effective method is considered to be the real-time detection of drunk drivers’ behaviors or drunk driving behaviors, which allows for further immediate interventions to ensure safety. This detection process generally adopts Deep Learning (DL) approaches, training Deep Neural Networks (DNNs) with collected drunk driving data [3,4]. DNNs learn to identify typical patterns—such as erratic lane weaving or gaze distraction—to detect potential drunk driving. It has been proven in many applications that given sufficient data, such data-driven methods can achieve satisfactory performance. However, drunk driving is an illegal activity that endangers public safety, making it virtually impossible to obtain large amounts of real-world drunk driving data. This limitation prevents drunk driving detection from replicating the successes found in previous research.

Research on drunk driving detection usually involves recruiting participants and deploying driving simulators in controlled, artificially constructed scenarios to gather drunk driving data. The substantial time and financial expenditures, along with the lack of diversity in the artificially designed scenarios, limit the amount and variety of data collected, making it insufficient to meet the data requirements for training supervised learning models and less reliable for evaluation purposes. A recent study [5] has shown the feasibility of detecting potential drunk driving behaviors in the same scenario using only raw normal driving data, thus considerably alleviating the data requirements for training. However, results obtained from a single scenario are insufficiently convincing. The performance under more complex and varied scenarios still requires further verification with larger datasets.

To advance toward practical applications, the next critical challenge lies in tackling more complex and diverse scenarios. To this end, we propose DUIncoder, which is an unsupervised framework designed explicitly for detecting drunk driving behavior in multiple driving scenarios. The main contributions of this paper are summarized as follows:

- We propose DUIncoder, which is a framework that requires only normal driving data for configuration and deployment. Compared to previous supervised learning detectors, DUIncoder not only eliminates the reliance on drunk driving data but also surpasses it in overall detection performance and generalization across different scenarios.
- In comparison to other unsupervised detectors that also rely solely on normal driving data, DUIncoder not only outperforms in terms of overall detection performance but also performs excellently in scenarios that challenge other methods. This underscores DUIncoder’s ability to learn from various scenarios and its superior generalizablity.
- Two versions of DUIncoder are proposed for different application scenarios. DUIncoderG is more lenient, requiring only a small amount of training data to be effective with a low false detection rate. DUIncoderR, on the other hand, is stricter, demonstrating better detection performance with larger amounts of data and a low missed detection rate.
- Experiments also demonstrate that as the available normal driving data increases, the performance of DUIncoder can be further improved. This reinforces the potential for DUIncoder to be further optimized in real-world applications by incorporating incrementally collected data particularly to handle corner cases more effectively.

The remainder of this paper is organized as follows: In Section 2, we provide a literature review on driver monitoring and DUI behavior detection. Section 3 details the methodology of DUI behavior detection, including the problem, approaches, and our proposed DUIncoder. The DUI dataset collected and utilized in this research is introduced in Section 4. In Section 5, we present the experiment setup, DUI detection results, detailed analysis, and ablation studies. We elaborate on our discoveries, the challenges encountered, and potential avenues for future research in Section 6. Finally, this paper concludes in Section 7.

## 2. Related Works

### 2.1. Driver State Monitoring

In driving, the driver is required to continuously monitor the surrounding environment, make appropriate decisions based on varying circumstances, and perform corresponding actions immediately, which is crucial for maintaining traffic safety [6]. Consequently, any deterioration in the driver’s state can significantly increase the risk of accidents [7]. For instance, a driver experiencing anger may process nearby traffic conditions more slowly and display an increased propensity for aggressive driving maneuvers [8].

Driver state monitoring aims to recognize negative driver states at an early stage to minimize the potential for traffic accidents [9,10]. The assessment of driver state primarily involves three dimensions. The first is physiological attributes, including features such as heart rate [11], pupil diameter [12], breathing activities [13], and brain activities [14]. Behavioral features, such as eye closure [15], blink duration [16], changes in gaze direction [17], and head movements [18] also serve as significant indicators. Furthermore, subjective measures, including the Karolinska sleepiness scale [19], Stanford sleepiness scale [20], visual analog scale [21], and the self-assessment manikin [22] are often incorporated into the evaluation of driver state.

While previous studies [23,24] have categorized driver states that are prone to causing traffic accidents into five categories—drowsiness, cognitive workload, distraction, emotion, and Driving Under the Influence (DUI)—only DUI can be directly defined through objective metrics such as Blood Alcohol Content (BAC). However, directly measuring a driver’s BAC typically requires additional devices (e.g., gas sensors), which introduce substantial costs and necessitate regular maintenance. The invasive process also relies on the driver’s cooperation—a requirement that is often challenging to meet in practice. Given the critical influence of driver state on traffic safety, developing reliable and non-invasive approaches for driver state detection is imperative.

### 2.2. Driving Under Influence Behavior Detection

DUI behaviors refer to a series of actions that, following alcohol intake, deviate from the driver’s typical behavior due to impaired nervous system function caused by increased BAC [25]. In this context, DUI behaviors are categorized into *DUI Driver Behavior* and *DUI Driving Behavior* based on the subjects and objects of behavioral manifestation.

*DUI Driver Behavior* refers to the driving-irrelevant actions of drivers after consuming alcohol, primarily involving body movements and facial expressions [26]. The detection of such DUI behaviors is achieved by using driver monitoring cameras to capture images from within the vehicle, which is followed by analyzing driver-specific behavior characteristics (e.g., gaze direction [27], aspect ratio of eyes [28] and mouth [29], lateral movement of body and head [30]) to identify particular intoxication patterns (e.g., head nodding or tilting, eye closing, yawning) [31] and further assess whether the driver is under the influence.

*DUI Driving Behavior* refers to the actions of a driver operating a vehicle after alcohol consumption or the resultant motion status of the vehicle. The detection of these behaviors is typically conducted via a Controller Area Network (CAN) or smartphones [32] to obtain features such as steering wheel angle, pedal position, vehicle speed, and rotation. These characteristics are then used to directly predict BAC [33] or to compare with expected drunk driving [34] or normal driving [5] behaviors to produce the final detection results.

While DUI has been a subject of public concern for several decades, most of the existing research concentrates on areas like drunk driving legislation, alcohol consumer surveys, or statistical analyses of the effects of alcohol on driving performance [3,23,35,36,37,38]. Research on detecting DUI behaviors, however, is still in its nascent stage. Current mainstream methods rely on techniques like Linear Discriminant Analysis (LDA) [39], Multi-Layer Perceptron (MLP) [40], Support Vector Machine (SVM) [4,41,42], Random Forest (RF) [34,43], and other machine learning approaches [44,45,46]. Recent studies have started to explore deep learning techniques such as Convolutional Neural Network (CNN) [33] and Variational Autoencoder (VAE) [5]. Despite achieving promising results in single scenarios, these approaches often fail to perform adequately in more complex and diverse environments.

## 3. Methodology

### 3.1. Problem Statement

The primary objective of this research is to mitigate traffic accidents associated with Driving Under Influence (DUI) to improve traffic safety. The immediate cause of these accidents is inappropriate “driving behavior” (as discussed in Section 2.2) exhibited by drunk drivers. Consequently, this study posits that achieving this goal requires a focus on driving behavior, particularly by addressing the following research problem:

**Problem** **1.** *How to determine whether a driver’s driving behavior is inappropriate?*

However, the term “inappropriate” is subjective, making it difficult to standardize and quantify. In contrast, “drunk” is a well-defined, objective, and quantifiable indicator (e.g., Blood Alcohol Content (BAC) exceeding 0.15 mg/L for breath or 0.3 mg/mL for blood in Japan). Within the context of this study, which considers only two statuses—“normal” and “drunk” —the following assumptions are introduced to establish a correspondence between “inappropriate” and “drunk”:

**Assumption** **1.** *If the driver is normal, then the corresponding driving behaviors are appropriate.*

**Assumption** **2.** *If the driver is drunk, then the corresponding driving behaviors are inappropriate.*

By introducing the aforementioned assumptions, a connection between DUI driving behavior and driver state is established, allowing for the assessment of driving behavior through an objective metric. The preceding Problem 1 can then be restated as Problem 2 and the solution can be formulated as Equation (1):

**Problem** **2.** *How can the driver’s status (normal or drunk) be inferred from the corresponding driving behaviors?*

(1) $${\hat{s}}_{t}=D\left(B_{t}\right)=\left\{\begin{array}{cc} 0 & \mathrm{if}\;\hat{s}\;\mathrm{corresponds}\;\mathrm{to}\;\mathrm{normal}\;\mathrm{status}\\ 1 & \mathrm{if}\;\hat{s}\;\mathrm{corresponds}\;\mathrm{to}\;\mathrm{drunk}\;\mathrm{status} \end{array}\right.$$

where the following apply:

- ${\hat{s}}_{t}\in \left\{0,1\right\}$ represents the predicted driver status at the moment *t*.
- D(B) is the detection algorithm to predict driver status from driving behaviors B.
- $B_{t}=\left\{b_{1},b_{2},\ldots ,b_{N}\right\}$ is the input consisting of *N* driving behavior features at the moment *t*.

In practice, the driver’s status ${\hat{s}}_{t}$ at a specific moment requires analyzing behaviors over the corresponding period. Since sudden shifts in the driver’s status are unlikely within a short time span, integrating the predicted status across the journey can yield more robust results. In summary, the detection of drunk driving behaviors in this study is formulated as follows:

(2) $${\hat{s}}_{t,W}=D\left(B_{t-\frac{W}{2},t+\frac{W}{2}}\right)=\left\{\begin{array}{cc} 0 & \mathrm{if}\;\hat{s}\;\mathrm{corresponds}\;\mathrm{to}\;\mathrm{normal}\;\mathrm{status}\\ 1 & \mathrm{if}\;\hat{s}\;\mathrm{corresponds}\;\mathrm{to}\;\mathrm{drunk}\;\mathrm{status} \end{array}\right.$$

(3) $${\hat{S}}_{t_{0},t_{N}}=agg\left({\hat{s}}_{t}:t\in \left\{t_{0},t_{1},\ldots ,t_{N}\right\}\right)$$

where the following apply:

- ${\hat{s}}_{t,W}\in \left\{0,1\right\}$ represents the driver status at the moment *t*, which is predicted based on data within a window size *W*.
- $B_{t-\frac{W}{2},t+\frac{W}{2}}$ is the input driving behavior features from moment $t-\frac{W}{2}$ to moment $t+\frac{W}{2}$.
- ${\hat{S}}_{t_{0},t_{N}}$ is the predicted driver status throughout the journey, spanning from moment $t_{0}$ to moment $t_{N}$.
- agg is the aggregation algorithm that integrates the frame-by-frame predictions of the driver’s status during the entire journey.

### 3.2. DUI Driving Behavior Detection

**Figure 1:**
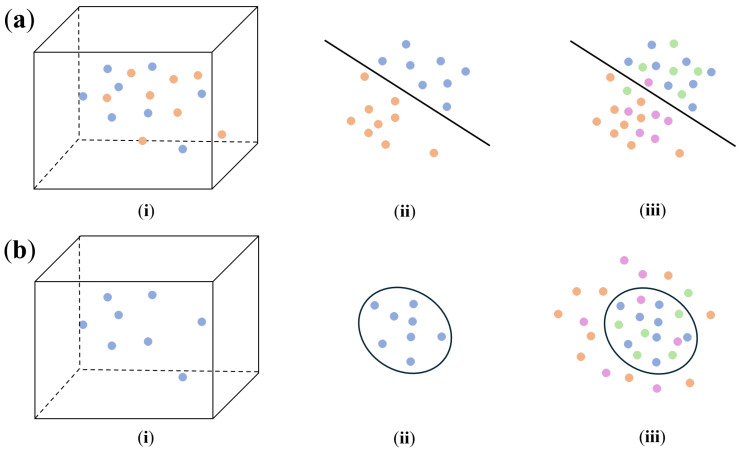
Illustration of two approaches for detecting DUI driving behaviors: (**a**) detect DUI driving behaviors using binary classification; (**b**) detect DUI driving behaviors using novelty detection.

The detection of DUI driving behaviors involves utilizing the driving behavior data to distinguish between normal and drunk driving during a journey. Depending on the training data requirements and methodology employed, the existing methods for DUI behavior detection [4,5,33,34,39,40,41,43,44,45,46] can be broadly categorized into two categories: *binary-classification-based* and *novelty-detection-based*. Figure 1 provides a summary of this process. In the figure, each point corresponds to a segment of driving behavior data over a specific time period. Points of the same color collectively form the driving behavior data for an entire journey with different colors signifying separate journeys. Blue and green point sets correspond to normal status, while orange and purple point sets correspond to drunk status.

*Binary-classification-based* driving behavior detection [4,33,34,39,40,41,43,44,45,46] frames the problem as a binary decision between normal and drunk driving. The process is depicted in Figure 1a: This approach typically requires (i) normal driving behavior data and drunk driving behavior data for training. Using either handcrafted or learned features, the input data are (ii) mapped from a high-dimensional space to a separable space, where a hyperplane is identified to partition the two categories. Later in the inference phase, new input data are also (iii) projected onto the same space and their classification is determined based on its position relative to the hyperplane.

*Novelty-detection-based* driving behavior detection [5] considers drunk driving as a novel status distinct from normal driving with the approach proposed in this paper also falling within this category. In Figure 1b, (i) only normal driving behavior is required during training. While leveraging similar approaches, it (ii) maps the data into a space that minimizes the dispersion of the point sets, allowing for the identification of a boundary hyperplane. For inference, new input data are (iii) projected into this space, and its classification is based on whether it lies inside or outside the defined hyperplane.

### 3.3. DUIncoder

In this work, we propose DUIncoder, which is a framework designed to detect drunk driving behavior across diverse driving scenarios using only normal driving data during training. The following sections provide a comprehensive explanation of DUIncoder.

#### 3.3.1. Overall Structure

**Figure 2:**
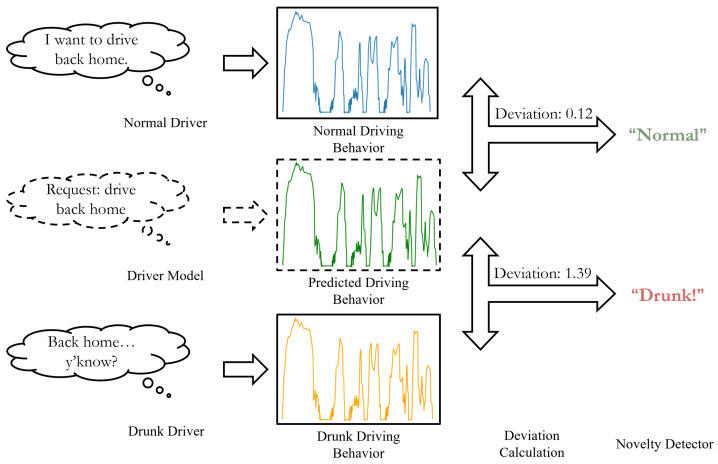
Illustration of the overall structure of DUIncoder.

The overall structure of DUIncoder is illustrated in Figure 2. The core principle of DUIncoder is to compare the driver’s observed behavior with the ideal driving behavior for a given scenario. The deviation between these behaviors is then analyzed to determine whether the driver is operating in a normal driving state.

To achieve this, normal driving behavior data from various scenarios are utilized to train a driver model capable of predicting the ideal driving behavior based on the driver’s observed behavior in any given scenario. The deviation between the observed and ideal behaviors is quantified using the L1 distance, and this deviation is subsequently fed into a novelty detector to determine whether the driver is operating in a normal state. Finally, the detection results from various segments of a journey are aggregated to produce a final determination of whether the driver was drunk during the journey.

#### 3.3.2. Driver Model

**Figure 3:**
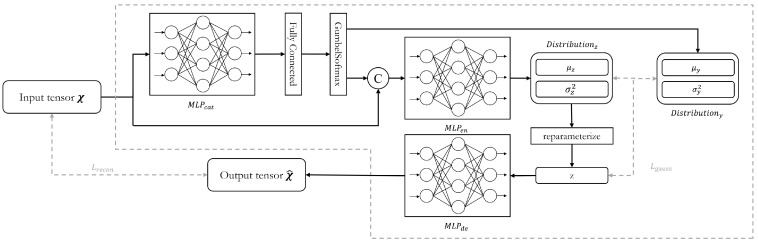
Network structure of the driver model in DUIncoder.

The purpose of the driver model is to generate the ideal driving behavior corresponding to the input driving behavior data from various scenarios. Recognizing that the data distributions of driving behaviors vary across scenarios and maneuvers, this challenge was addressed by incorporating insights from prior research [5,47,48] to inform the network design. The structure of the network is illustrated in Figure 3.

The raw driving behavior data are first sampled using a sliding window centered at moment *t*, which is followed by a z-score normalization. After being flattened, the data are input into the driver model as a tensor (x) of shape [B,N×L], where *B* denotes the batch size, *N* indicates the number of selected driving behavior features, and *L* represents the size of the sliding window.

In the driver model, the first operation is the extraction of categorical features from the input tensor. This process involves a Multi-Layer Perceptron $MLP_{cat}$, a fully connected layer, and a GumbelSoftmax layer. The $MLP_{cat}$ is composed of multiple fully connected layers, which are each followed by a LeakyReLU activation. The features output by $MLP_{cat}$ are weighted and combined through a fully connected layer and then passed through the GumbelSoftmax layer. This layer samples from the categorical distribution to assign distinct categorical features to each element of the feature dimension, resulting in a tensor of shape $\left[B,N_{cat}\right]$. This categorical feature tensor is used to produce the mean tensor ($\mu_{y}$) and the variance tensor ($\sigma_{y}^{2}$) of the prior latent distribution. It is also concatenated with the input tensor and fed into the subsequent network. The concatenated tensor is then passed through the $MLP_{en}$ encoder, producing the mean tensor ($\mu_{z}$) and the variance tensor ($\sigma_{z}^{2}$) of the generator latent distribution. Next, reparameterization is applied, and random samples are drawn from this distribution to produce the sample tensor (z). This sample tensor is then used to generate the reconstruct tensor ($\hat{x}$) of shape $\left[B,N\times L^{\prime }\right]$ by the $MLP_{de}$ decoder.

During training, the driver model performs random sampling from the latent distribution and computes the reconstruction loss $L_{\mathrm{recon}}$ and Evidence Lower Bound (ELBO) loss $L_{\mathrm{gauss}}$ for backpropagation. The total loss L used in the model is defined as follows:

(4) $$L=L_{\mathrm{recon}}+L_{\mathrm{gauss}}$$

(5) $$L_{\mathrm{recon}}=∥x_{t-\frac{L^{\prime }}{2},t+\frac{L^{\prime }}{2}}-{\hat{x}}_{t-\frac{L^{\prime }}{2},t+\frac{L^{\prime }}{2}}∥$$

(6) $$L_{\mathrm{gauss}}=\log p\left(z|\mu_{z},\sigma_{z}^{2}\right)-\log p\left(z|\mu_{y},\sigma_{y}^{2}\right)$$

(7) $$\log p\left(x|\mu ,\sigma^{2}\right)=-\frac{1}{2}\left[\log\left(2\pi \right)+\log\left(\sigma^{2}\right)+\frac{{\left(x-\mu \right)}^{2}}{\sigma^{2}}\right]$$

#### 3.3.3. Implementation Process

**Figure 4:**
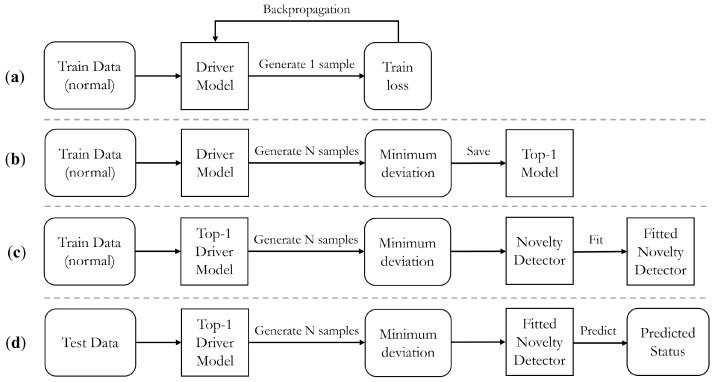
Illustration of the implement process of DUIncoder: (**a**) training process of the driver model; (**b**) validating process of the driver model; (**c**) fitting process of the novelty detector; (**d**) inferring process of DUIncoder.

As DUIncoder consists of various modules that must be trained asynchronously, Figure 4 illustrates the distinct stages of its implementation, providing a comprehensive overview of the entire process. The implementation begins with training the driver model, as depicted in Figure 4a. This procedure, previously outlined in Section 3.3.2, involves utilizing normal driving behavior data, sampling from the latent space during each iteration, generating a sample, and calculating the loss function for subsequent backpropagation.

During training, a distinct validation procedure is adopted, as shown in Figure 4b. In this stage, a sufficient number of instances are sampled from the latent distribution. The frame-wise minimum deviation from the input tensor is calculated and subsequently used for selecting the top-performing model during training.

Once the training of the driver model is complete, the same normal driving behavior data are utilized to fit the novelty detector. This process, illustrated in Figure 4c, involves calculating the minimum deviation, which serves as the input to the novelty detector. The novelty detector leverages the minimum deviation input to calculate the deviation boundary in normal status.

Finally, the inferring process is shown in Figure 4d. Deviation between the observed behavior and the ideal driving behavior is calculated and utilized for determining the status of the driver.

## 4. DUI Dataset

### 4.1. Simulator Setup

**Figure 5:**
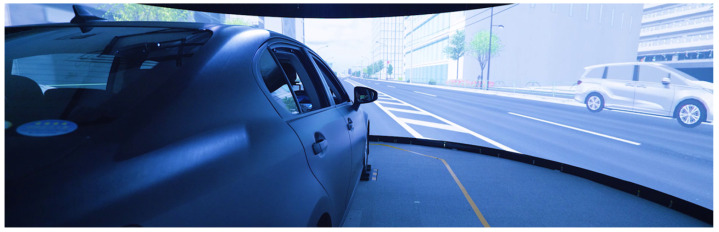
The driving simulator for collecting both normal and drunk driving behaviors.

In line with our previous research [5], a DUI dataset is constructed using a simulator for training and evaluating the proposed approach. The driving simulator at the Advanced Research and Innovation Center, DENSO CORPORATION, was employed to collect data for this study. As illustrated in Figure 5, the simulator features six screens to provide a 360-degree panoramic view. The cabin, modeled after an actual vehicle, allows for three Degrees of Freedom (DoF) in motion, including pitch, roll, and heave. The simulator captures multiple data streams: vehicular information from the CAN bus, internal and external video feeds from driver monitoring and drive recorders, electrocardiogram signals via Silmee, and pulse measurements via Fitbit.

### 4.2. Scenario Design

The primary objective of this study is to identify drunk driving incidents in realistic driving scenarios with a particular focus on the Japanese driving contexts. To achieve this, a simulated environment was constructed based on the traffic infrastructure surrounding Shinagawa Station in Tokyo. Within this simulation, three distinct routes were designed to capture driving behaviors under various circumstances.

**Figure 6:**
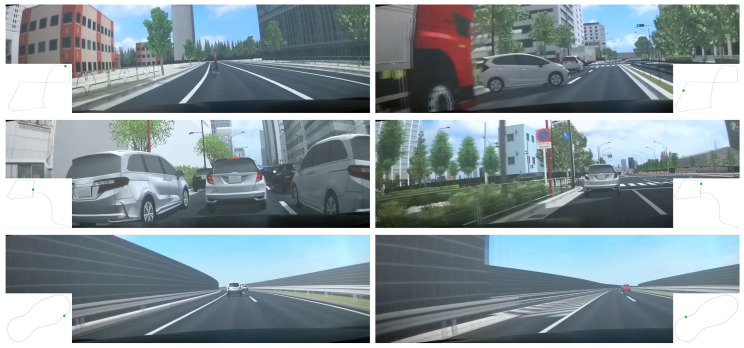
Examples of scenes in the three different routes: ((**top**): route “near accident urban”; (**middle**): route “accident”; (**bottom**): route “highway”). The green point in the trajectory maps indicates the vehicle’s current location.

The three routes, as illustrated in Figure 6, are designated as “near accident urban” (hereafter referred to as “accident”), “urban” and “highway”. Route “accident” simulates a typical Japanese urban roadway, incorporating potential accident scenarios such as sudden lane changes by vehicles in adjacent lanes, pedestrians intruding onto the roadway, and vehicles emerging from blind spots. Route “urban” represents a similar environment to Route “accident” but with a more stable traffic flow. It primarily involves scenarios like car following within urban roads. In contrast, Route “highway” depicts a Japanese highway, which is characterized by lower traffic density and higher speeds compared to Routes “accident” and “urban”.

### 4.3. Data Collection

A total of 11 participants are involved in the data collection, all of whom are male with ages ranging from their 20s to 60s. All participants held valid Japanese driver’s licenses. The participants’ driving and drinking-related characteristics are detailed in Table 1.

**Figure 7:**
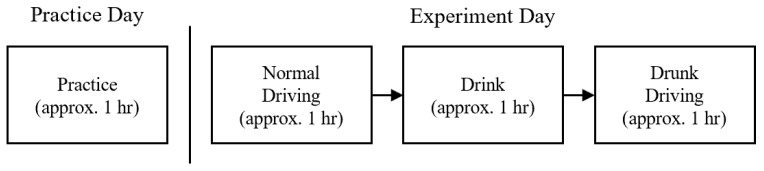
Flow diagram of the data collection process.

As shown in Figure 7, all participants first underwent approximately one hour of pre-experiment practice to familiarize themselves with the simulator. On the experiment day, an initial hour was devoted to collecting normal driving data, during which each instance of driving on every route was saved as a separate driving record. This was followed by an approximately one-hour interval for drinking, during which participants consumed 2–3 units of sake according to their body weight to achieve a BAC level of 0.40 mL/L in breath [2]. Following alcohol consumption, participants ingested a small amount of water and waited for 10 min to mitigate residual alcohol effects in the oral cavity that might influence the results. Measurements were taken at the beginning and end of the drinking phase to ensure achieving the desired BAC level. Finally, there was approximately one hour allocated for the drunk driving, during which the driving scenario replicates that of the preceding normal driving.

Given the involvement of human subjects, this research obtained approval for all ethical and experimental procedures from the respective Ethics Committees of DENSO CORP and Nagoya University.

### 4.4. Driving Behavior Data

As discussed in Section 3.1, the detection of DUI driving behaviors involves predicting the driver’s status over a defined time interval based on the temporal driving behavior data. In this study, the term “driving behavior” specifically refers to the manipulation of the vehicle and the resulting changes in the vehicle’s motion state.

**Figure 8:**
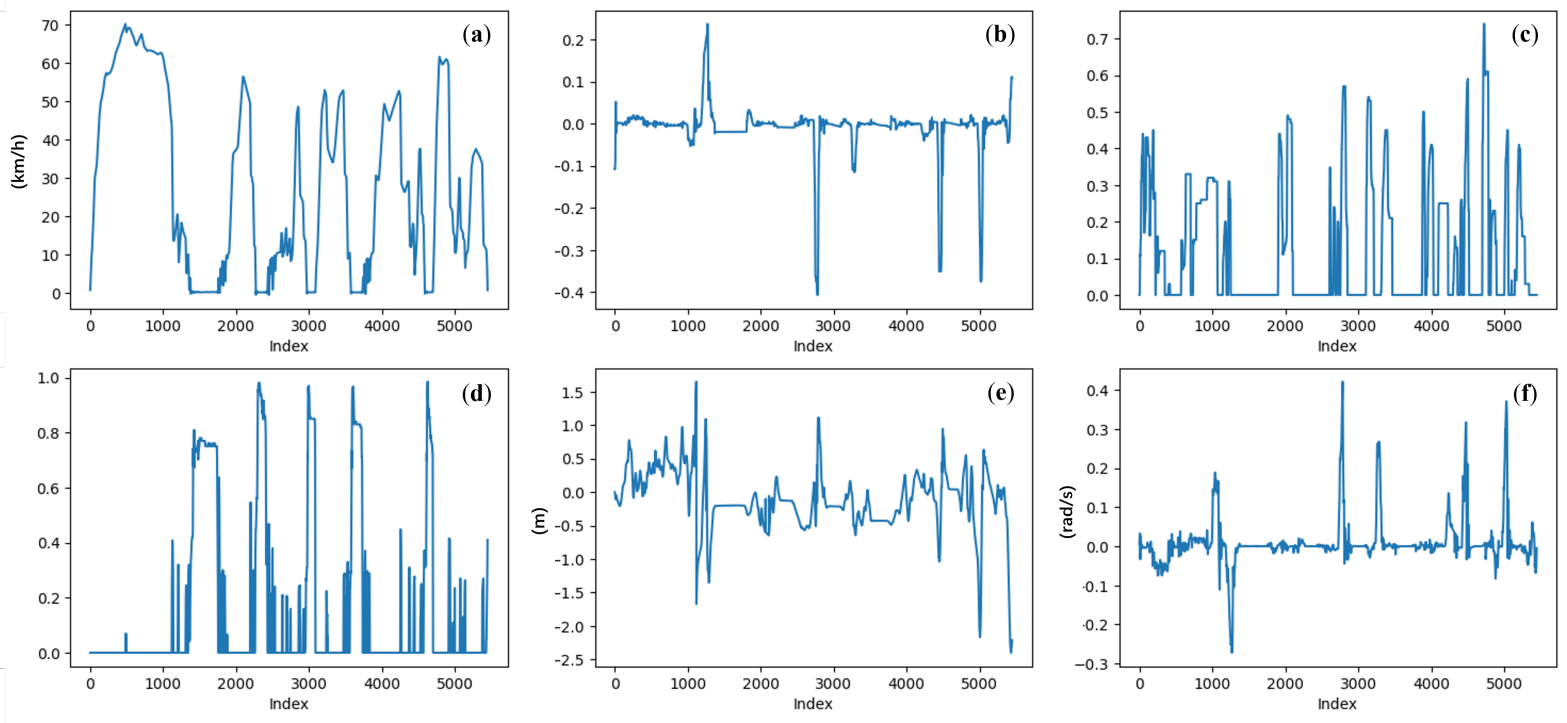
Example of driving behaviors during a journey: (**a**) vehicle velocity (km/h); (**b**) steering angle ([−1, 1]); (**c**) throttle position ([0, 1]); (**d**) brake position ([0, 1]); (**e**) lane center deviation (m); (**f**) yaw rotation speed (rad/s).

Figure 8 illustrates the driving behavior data during a journey. Figure 8b–d correspond to the driver’s manipulations, while Figure 8a,e,f correspond to the vehicle’s motion state. By integrating these two types of features, a comprehensive depiction of the driving scenario is achieved, addressing both the causes (e.g., a sudden change in throttle input) and the outcomes (e.g., excessive vehicle acceleration). This integration facilitates the reasonable inference of the driver’s state during the observed period.

## 5. Experimental Evaluation

### 5.1. Evaluation of DUI Driving Behavior Detection

To validate the performance of DUIncoder, we conducted an evaluation of its effectiveness in detecting DUI driving behavior in different scenarios. The following sections provide a comprehensive description of the experimental process.

#### 5.1.1. Experiment Setup

For a comprehensive evaluation of DUIncoder’s effectiveness in detecting drunk driving behavior across diverse driving scenarios and maneuvers, we conducted an assessment using all three routes from the DUI dataset. Due to the observed hindrance of KL divergence in minimizing reconstruction error during training [5,49], and considering the distinction between the DUI detection task and traditional generative tasks, in the experiment, we proposed and evaluated two versions of DUIncoder:

- *DUIncoderG*: focuses on generating a broader range of normal driving behaviors by utilizing both the reconstruction loss $L_{\mathrm{recon}}$ and the ELBO loss $L_{\mathrm{gauss}}$, as discussed in Section 3.3.2.
- *DUIncoderR*: emphasizes reconstructing normal driving behavior specific to the present scenario by using only the reconstruction loss $L_{\mathrm{recon}}$ during training.

Aside from the loss function, the network structures of the two DUIncoders are implemented identically with PyTorch [50] (Version 1.12.1). An isolation forest, implemented with scikit-learn [51] (Version 1.3.0), was used for novelty detection with the contamination parameter configured to 0.01.

Several baseline methods were selected for comparison following the previous study [5], including Linear Discriminant Analysis (LDA) [39], Adaptive Boosting (AdaBoost) [43], Extreme Gradient Boosting (XGBoost) [52] (Version 2.0.3), Support Vector Machine (SVM) [4], Random Forest (RF) [34], and Variational Autoencoder (VAE) [5]. The details of implementations are mentioned in Table 2. The choice of hyperparameters was made by considering both previous studies [4,5] and our own practice. To optimize performance, certain classifiers were implemented using cuML [53] (Vesion 24.10.00) as an alternative framework. VAE incorporated the same isolation forest, with contamination set to 0.01, for novelty detection to align with DUIncoders.

We incorporated insights from previous studies [5,30] alongside the information accessible from the simulator to comprehensively capture driving behaviors. Six key features were selected as raw inputs, including vehicle speed, steering angle, throttle position, brake position, lane center deviation, and yaw rotation speed. The entire DUI dataset was divided into training, validation, and test sets in a ratio of 7:2:1 with driving records serving as the unit of segmentation. The supervised learning classifiers (LDA, AdaBoost, XGBoost, SVM, RF) were trained using normal driving data and an equal amount of drunk driving data, while the VAE and DUIncoder used only the normal driving data, which were also employed during the fitting of the isolation forest. A sliding window of 10 s (101 frames of data) was applied to extract driving behavior samples that contain temporal information from each driving record. Samples at the record boundaries were padded using edge padding. In a normal driving record, all frames were considered instances of normal behavior, while in a drunk driving record, the frames were similarly classified. Finally, z-score normalization was adopted for each driving behavior feature, using the mean value of (55.372,−0.004,0.178,0.070,−0.090,0.003) and standard deviation of (19.734,0.069,0.145,0.240,0.470,0.066).

Section 3.3.3 outlines the implementation steps of DUIncoder. The driver model was trained for 2000 epochs on the training set. In the experiments, we used Adam [54] as the optimizer to train the driver model. The initial learning rate was set to 0.0004 with a decay rate of 0.95 per 50 epochs. For DUIncoderG, a cosine annealer [49] with the period of 200 epochs was applied to prevent the ELBO from vanishing during training. At intervals of 20 epochs, the validation process was performed; 32 instances were sampled from the latent space to calculate the frame-wise minimum deviation. Training and validation loss curves for driver models in DUIncoders are illustrated in Appendix A. The same number of 32 instances was also used for sampling in both the novelty detector fitting and DUIncoder inferring processes.

#### 5.1.2. Overall Detection Result

**Figure 9:**
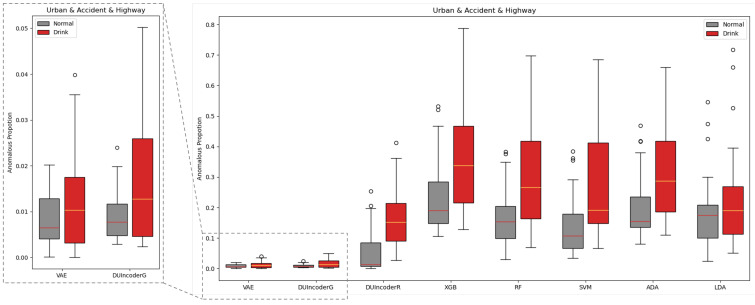
Box-plot comparison with baseline methods in terms of detecting DUI driving behavior in normal and drunk records across all three different routes.

An evaluation of DUIncoders and other baseline methods was conducted on the test set, where all three routes are included. For each driving record in the test set, the anomalous proportion (AnoP) was computed. AnoP is defined as the ratio of detected anomalous frames to the total number of frames. The AnoP values for records with different driver statuses detected by various methods were aggregated and visualized in a box plot, as shown in Figure 9.

For a more comprehensive analysis, Table 3 presents the statistical metrics for each boxplot, including the maximum, minimum, median, and Interquartile Range (IQR), which were calculated after removing outliers. In addition, the Area Under the Curve (AUC) of the Receiver Operating Characteristic (ROC) curve and the *p*-value were chosen as performance metrics. The AUC evaluates the normal–drunk classification performance of each method in distinguishing driving records based on AnoP with higher AUC values indicating better classification performance. The *p*-value is derived from a T-test performed on the AnoP values for the normal and drunk driving groups and is used to assess whether the differences between the two distributions are statistically significant. A smaller *p*-value indicates a lower likelihood that the observed differences occurred by chance with values below 0.05 or 0.01 typically considered statistically significant.

In general, for all methods, the AnoP values in the normal group were consistently lower than those in the drunk group. The binary classification-based supervised learning classifiers produced relatively high AnoP values for both groups, with the median AnoP in the normal group ranging between 0.10 and 0.19, suggesting that 10% to 19% of normal driving is falsely detected as impaired driving. In contrast, DUIncoders and VAE resulted in significantly lower AnoP values in both groups, with the median AnoP in the normal group remaining close to 0.01, which was consistent with the contamination rate of 0.01 setting in the isolation forest.

Specifically, in terms of AUC and *p*-value, DUIncoderR demonstrated the best performance across all methods with an AUC of 0.847 and a *p*-value below 0.001. DUIncoderG, trained solely on normal driving behavior data, achieved an AUC of 0.657, slightly lower than the performance of supervised classifiers, which ranged from 0.750 to 0.760. The *p*-value for DUIncoderG was 0.004, which was comparable to that of the supervised learning classifier. However, both AUC and *p*-value metrics for DUIncoderG still outperformed those of VAE, which was also trained using only normal driving data.

**Figure 10:**
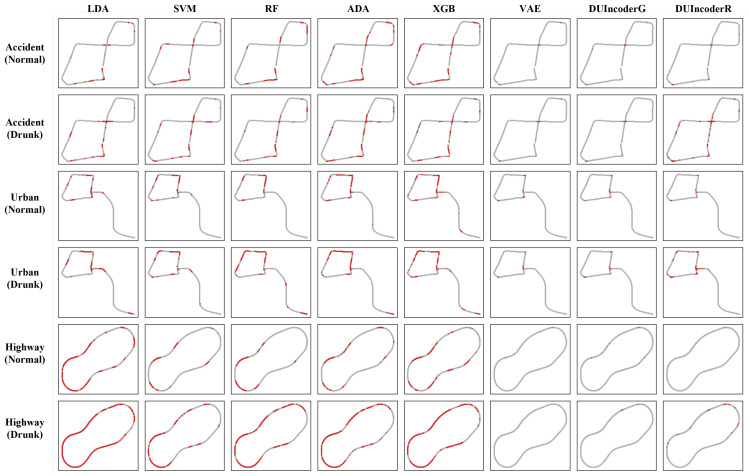
Trajectory comparison with baseline methods in terms of detecting DUI driving behavior in normal and drunk records across all three different routes. The red points in the trajectory denote frames predicted as drunk driving.

To provide a more intuitive comparison of the detection results, a driver’s normal and drunk driving records from the same day in the test set were selected and visualized as a trajectory plot. The resulting trajectory visualization is presented in Figure 10.

In the normal group, potential misdetections by DUIncoderR often align with misdetections made by other supervised classifiers, though the number of misdetections is substantially higher for the supervised classifiers. This observation suggests that DUIncoderR does not introduce additional misdetections compared to other supervised learning classifiers.

When comparing DUIncoderR with DUIncoderG and VAE, it is noted that while DUIncoderG and VAE exhibit fewer potential misdetections, these misdetections often occur at similar locations. This indicates that such misdetections may be attributable to the driver committing minor inappropriate driving behaviors at specific locations, even during normal driving.

For the drunk group, DUIncoders detect fewer anomalous frames compared to the supervised classifiers. However, these frames are predominantly located in critical areas, such as turns, which are regions where drunk driving behaviors are more likely to manifest and increase the risk of accidents.

#### 5.1.3. Visualization and Explanation

**Figure 11:**
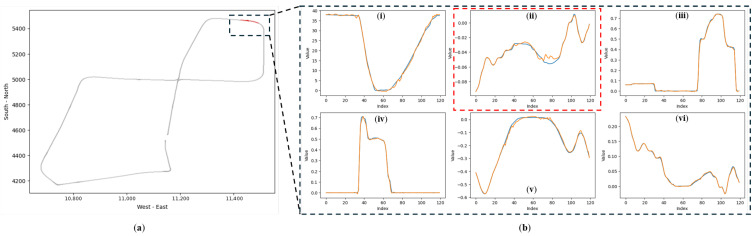
Comparison of actual and predicted driving behaviors in anomalous segments detected by DUIncoderR in “accident” route: (**a**) the red section within the dashed box indicates the location of the selected detected anomalous segment within the entire trajectory; (**b**) a comparison of actual and predicted driving behaviors within this segment, where orange represents the predicted driving behavior, and blue represents the actual driving behavior: (**i**) vehicle velocity (km/h); (**ii**) steering angle ([−1, 1]); (**iii**) throttle position ([0, 1]); (**iv**) brake position ([0, 1]); (**v**) lane center deviation (m); (**vi**) yaw rotation speed (rad/s).

Unlike conventional supervised classifiers, DUIncoder generates the anticipated driving behavior for the current scenario, enabling interpretation of the inferred driver status. Figure 11 illustrates a segment of a drunk driving record selected from the test set.

The selected segment consists of approximately 130 frames, corresponding to a duration of around 13 s. While the actual driving behavior is generally consistent with the predicted behavior during this period, deviations occur within specific intervals (highlighted by the dashed box in Figure 11b(ii), where the actual steering wheel angle is larger than the expected value. This suggests that the driver exhibited larger steering angles and faster steering movements than expected, resulting in the detection of a drunk status for this segment.

### 5.2. Ablation Studies

To gain a deeper understanding of DUIncoder, a series of ablation experiments were conducted, focusing on various aspects of its functionality. These experiments are detailed in the following sections.

#### 5.2.1. Scenario-Wise Detection Analysis

**Figure 12:**
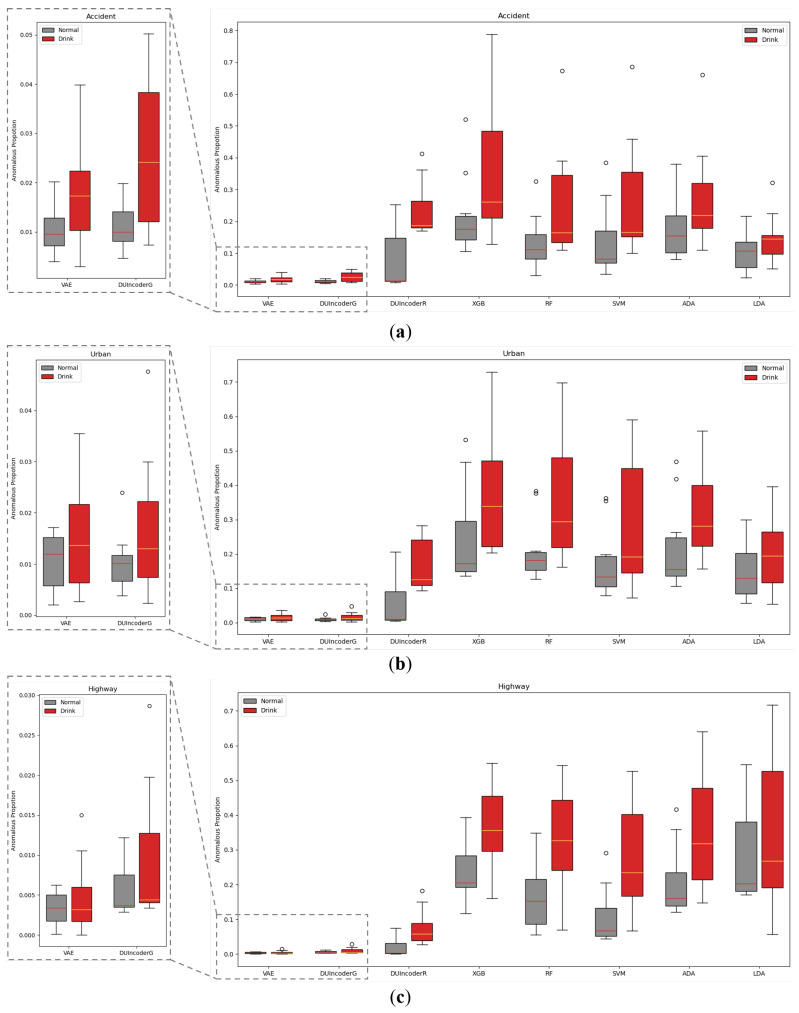
Box-plot comparison with baseline methods in terms of detecting DUI driving behavior in normal and drunk records of three different routes, respectively: (**a**) route “accident”; (**b**) route “urban”; (**c**) route “highway”.

In Section 5.1.2, we evaluated the overall detection performance of various methods across three different scenarios. To further analyze performance within each individual scenario, we conducted a separate evaluation of each method within each scenario. The AnoP values for records in each scenario with drivers of different status detected by various methods were aggregated and visualized in a box plot, as shown in Figure 12. The corresponding performance metrics are summarized in Table 4.

In general, DUIncoderR stayed consistent with previous results, demonstrating superior performance with an AUC value around 0.870 and *p*-value around 0.01 across different scenarios. Its AUC surpassed the second-best method by 0.056, 0.089, and 0.034 in the accident, urban, and highway scenarios, respectively. DUIncoderG’s performance in the accident scenario was only marginally below that of DUIncoderR, and in the urban and highway scenarios, it performed similarly to other supervised learning approaches. Most supervised classifiers maintained consistently strong performance across all scenarios with AUC values around 0.750 to 0.780 in different scenarios. Among them, SVM’s performance demonstrated greater fluctuation: it performed somewhat below average in the urban scenario while outperforming the average in the highway scenario. These fluctuations can be explained by SVM’s greater AnoP variance for the drunk group in urban and its lower mean AnoP for the normal group in the highway scenario. Meanwhile, LDA performed below average, maintaining an AUC of around 0.600 across the scenarios. VAE demonstrated solid performance in the accident scenario, but it failed to maintain consistent performance in other scenarios. Across all scenarios, its results were inferior to those of both DUIncoderR and DUIncoderG.

It is worth noting that in the highway scenario, VAE’s AUC of 0.5 suggests its performance was equivalent to random guessing in this case. However, DUIncoderG, despite also being a generative-oriented approach, exhibited much better performance than VAE in the highway scenario, underscoring the ability of DUIncoder to perform robustly in a variety of scenarios.

#### 5.2.2. Introducing of Data from Additional Scenarios

**Figure 13:**
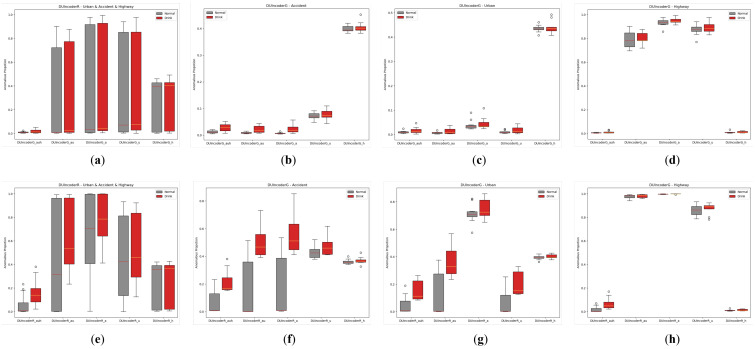
Box-plot comparison of DUI behavior detection by DUIncoders trained on different scenarios in normal and drunk data for three routes: (**a**) comparison between DUIncoderGs on all three routes; (**b**) comparison between DUIncoderGs on route “accident”; (**c**) comparison between DUIncoderGs on route “urban”; (**d**) comparison between DUIncoderGs on route “highway”; (**e**) comparison between DUIncoderRs on all three routes; (**f**) comparison between DUIncoderRs on route “accident”; (**g**) comparison between DUIncoderRs on route “urban”; (**h**) comparison between DUIncoderRs on route “highway”.

In Section 5.2.1, it was observed that many methods struggled to achieve consistent performance across different scenarios with VAE’s performance in the highway scenario being a notable example. This challenge may arise from significant differences in driving behavior distributions across scenarios, particularly between urban and highway environments, which complicates the simultaneous learning of both distributions. DUIncoder, however, demonstrated superior consistency across scenarios. To further investigate the effects of multi-scenario data on DUIncoder, we trained DUIncoderG and DUIncoderR with data from various scenarios and evaluated their performance in different settings. The AnoP distribution and performance metric are presented in Figure 13 and Table 5.

From Table 5, it can be observed that DUIncoders achieve enhanced overall performance as the number of scenarios used during training grows. In individual scenarios, performance either remains stable or improves with the inclusion of more scenarios in training. This indicates that DUIncoders can effectively address the problem of learning from multiple distinct data distributions.

Additionally, as shown in Figure 13, although DUIncoderR delivers strong results across all metrics, it produces a high AnoP for both the normal and drunk groups when encountering scenarios significantly different from the training data, such as highways. In some cases, the AnoP approaches 1, indicating a high rate of false detections under these conditions. In contrast, while DUIncoderG performs comparably worse across most metrics, it does not produce as many false detections under the same conditions. This suggests that DUIncoderG adopts a more conservative approach to generalization, which may be advantageous in situations where minimizing false detections is critical, such as ensuring a smooth driving experience.

Notably, when trained exclusively on highway data, DUIncoders faced challenges not only in generalizing to other scenarios but also in performing poorly in the highway scenario. This may be attributed to the limited diversity of driving behaviors in the highway scenario and the highly imbalanced data distribution. This underscores the importance and necessity of introducing data from a variety of different scenarios.

#### 5.2.3. Size of the Sliding Window

**Figure 14:**
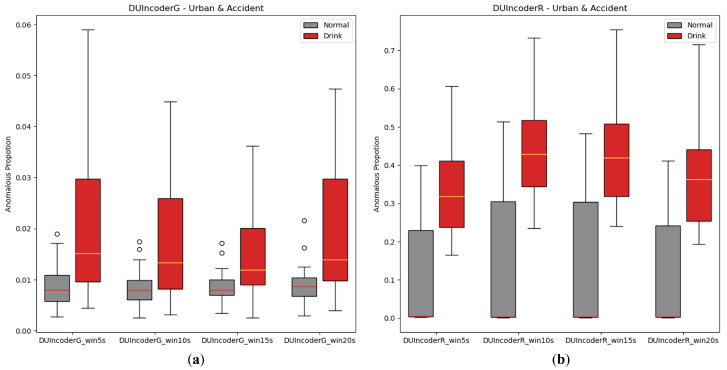
Box-plot comparison of DUI behavior detection by DUIncoders using different size of sliding window for route “accident” and route “urban”: (**a**) different window size setting for DUIncoderG; (**b**) different window size setting for DUIncoderR.

In the experiments presented in this paper, we followed the settings from prior studies [5], using a 10-s sliding window to extract samples from driving records. Given that larger sliding windows are commonly believed to provide improved contextual information, additional experiments were conducted with DUIncoders to evaluate the influence of sliding window size. In the experiment, the sizes of the sliding window from 5 s to 20 s are chosen for comparison. The AnoP distribution and performance metrics are illustrated in Figure 14 and Table 6. As mentioned in Section 5.2.2, the data distribution of the route “highway” might differ significantly from that of the route “accident” and the route “urban”, which may impact the experimental results. Therefore, all training and testing in the subsequent ablation studies were conducted exclusively in the route “accident” and the route “urban”.

As shown in Table 6, when the sliding window size increases, such as from 10 s to 20 s, both the AUC and *p*-value exhibit a slight improvement, indicating that larger sliding windows do have a positive impact on DUIncoders’ detection performance. However, this improvement is marginal and almost negligible. Increasing the sliding window size also results in higher computational and memory overhead, in addition to affecting real-time performance, which can sometimes be critical. Therefore, for DUIncoder, the sliding window size is not a decisive factor, and the optimal size may vary depending on the specific requirements and constraints of the application.

#### 5.2.4. Hyperparameter of Novelty Detector

**Figure 15:**
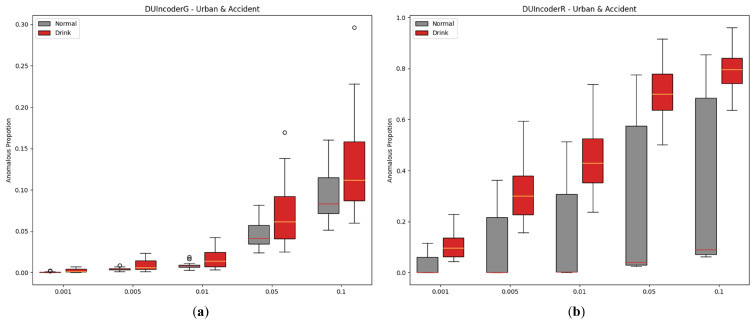
Box-plot comparison of DUI behavior detection by DUIncoders using different hyperparameter settings of novelty detector for route “accident” and route “urban”: (**a**) different settings for DUIncoderG; (**b**) different settings for DUIncoderR.

In DUIncoder, a novelty detector is employed to learn the boundaries of normal group data, enabling the identification of drunk driving behaviors. Since noise points may be present in normal data, a hyperparameter is leveraged to filter out noise, representing the fraction of potential anomalies within the normal data. In this study, the novelty detector employs an isolation forest with the contamination parameter set to 0.01 during experiments. To analyze the influence of this hyperparameter on detection performance, configurations from 0.001 to 0.01 were evaluated. The results are displayed in Figure 15 and Table 7.

From the experimental results, it can be observed that increasing the contamination parameter leads to a slight decline in performance for DUIncoderG. Conversely, DUIncoderR shows a slight improvement. However, a more critical observation for DUIncoderR is the AnoP distribution depicted in the figure: as contamination increases, the AnoP for the normal group escalates rapidly, resulting in a substantial number of false detections that far exceed the configured contamination value, which is undesirable. In summary, a higher contamination setting enables the detection of more anomalous events but at the cost of increased false detections. On the other hand, lower contamination settings result in fewer false detections but increase the missed detection rate for the drunk group, reducing the detectable drunk driving behaviors and, in extreme cases, causing them to vanish entirely.

#### 5.2.5. Increment of Normal Data

**Figure 16:**
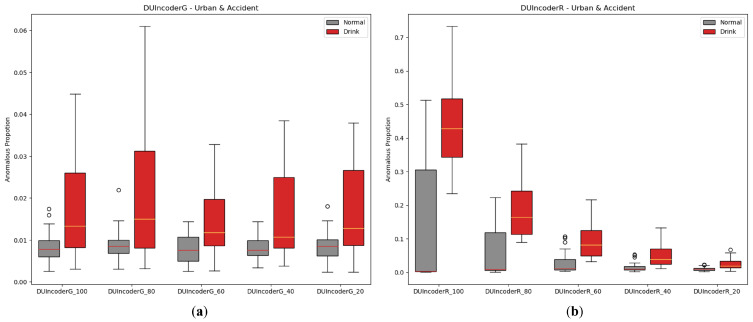
Box-plot comparison of DUI behavior detection by DUIncoders using different proportion of training data for route “accident” and route “urban”: (**a**) different settings for DUIncoderG; (**b**) different settings for DUIncoderR.

One of DUIncoder’s core objectives is to minimize its dependency on the quantity and variety of training data, enabling it to continuously improve performance by utilizing large volumes of normal data from diverse scenarios that can be readily obtained in future real-world applications. To simulate the process of incremental data availability, we performed experiments by randomly extracting subsets of the training data, ranging from 20% to 100% in five steps. The experimental results are depicted in Figure 16 and Table 8.

The results show that for DUIncoderG, the AUC remains around 0.7 as the data proportion increases from 20% to 100%, indicating that DUIncoderG can effectively learn key information from a small amount of data to enable stable performance from the beginning. In contrast, DUIncoderR demonstrates a significant improvement in detection performance as training data increases, suggesting that DUIncoderR has the potential to achieve higher performance as the available data become more diverse and abundant.

#### 5.2.6. Memory Consumption and Inference Time

As DUIncoder is intended for deployment in vehicles to detect DUI driving behavior, its memory consumption and inference time are also pivotal. Accordingly, we evaluated its performance across various devices. Since DUIncoderR and DUIncoderG share the same model architecture, we selected only DUIncoderR for assessment with the experimental results presented in Table 9.

Regarding memory consumption, DUIncoder typically occupies around 320 MB, which poses no substantial burden for contemporary computing systems. The inference time varies significantly with device performance, ranging from 1100 ms to 1800 ms, seemingly falling short of real-time processing. However, this is primarily due to our current implementation still relying on a sequential approach to sample a sufficient number of instances (as mentioned in Section 5.1.1, 32 instances are sampled to calculate the frame-wise minimum deviation). With future engineering optimizations, the reaction time could theoretically be reduced by approximately 30 times through parallel processing, bringing it down to around 35~60 ms, which would be sufficient to meet the requirements for practical deployment.

## 6. Discussion

In this paper, we proposed two versions of DUIncoder: DUIncoderG, which focuses on generating diverse normal driving behaviors, and DUIncoderR, which focuses on generating the most appropriate normal driving behavior for the current scenario. We find that these two approaches are complementary to some extent. DUIncoderG adopts a more lenient detection strategy and achieves excellent results with less data, while DUIncoderR applies a stricter detection approach and demonstrates further performance improvements with additional data. Combining these two approaches in practical applications may further enhance detection performance.

Nevertheless, there are still limitations within the current DUIncoder. At present, DUIncoder utilizes only driving behavior data, which, while closely linked to accidents, offers a limited perspective. Driving is a complex process influenced by numerous variables. Under different objective conditions such as traffic light states or road signs, and subjective conditions such as driver characteristics and intentions, identical behaviors (e.g., stopping at an intersection) may be evaluated entirely differently (e.g., stopping at a red light is appropriate, whereas stopping at a green light is generally not). Therefore, relying solely on driving data without accounting for these influential conditions might result in an incomplete description of the driving context, diminishing the credibility of the results. It is essential to integrate multimodal data (e.g., camera images, LiDAR point clouds, high-definition maps, thermal images, etc.) in order to achieve a comprehensive depiction of the current driving scenario and to further enable a reasonable assessment of the appropriateness of driving behavior.

Another problem is that, as frequently mentioned in Section 5, both DUIncoderG and DUIncoderR encounter unavoidable issues of false and missed detections. We believe that these issues are connected to Assumption 1 and Assumption 2 outlined in Section 3.1, which are also mentioned in previous research [5], pointing out that driving behaviors in a normal status are not always appropriate and vice versa. Since the objective of this research is to facilitate future enhancements to the model using raw driving data collected from real-world scenarios, introducing additional labeling would impose a considerable burden, which runs counter to the foundational aim of the study. Recent advancements in Large Language Models (LLMs) [55,56,57] have not only shown unprecedented performance and generalizability across a wide range of natural language tasks but also provided valuable commonsense knowledge that is able to combine with other modalities like Vision-Language Model (VLM) [58,59,60,61] and further significantly benefit other domains [62,63,64]. Particularly in the driving domain, human driving behaviors are derived from a coherent cognitive and logical flow, while LLMs are capable of similar reasoning [65]. The generalizable commonsense knowledge and logical reasoning abilities akin to humans allow LLMs to effectively transfer to the driving domain at minimal cost [66]. LLMs may be capable of replacing subjective human labeling, providing accurate assessments of driving behavior to eliminate approximation errors introduced by assumptions. Alternatively, a more advanced approach could involve distilling a lightweight, specialized LLM to directly and in real time detect inappropriate driving behaviors. However, even with the assistance of LLMs, this process still undoubtedly requires a significant amount of time and computational resources. A current feasible approach might be to strike a balance between safety (minimizing missed detections) and user comfort (reducing false detections).

## 7. Conclusions

Our study aims to mitigate traffic accidents caused by drunk driving. Given that existing methods often depend on difficult-to-acquire drunk driving data, we proposed DUIncoder, which is a framework designed to detect drunk driving behaviors by learning from normal driving data. DUIncoder seeks to enhance detection performance in real-world scenarios through the continuous acquisition of extensive and diverse normal driving data. Currently, DUIncoder surpasses supervised detectors that rely on drunk driving data even when only normal driving data are used. Experimental results show that DUIncoder demonstrates strong generalization across various driving scenarios and achieves further improvements in detection performance with incremental data, meeting the expectations for its future practical applications. In the future, we plan to incorporate multimodal driving data and integrate commonsense knowledge from approaches such as Large Language Models (LLMs) to further improve the representation of driving scenarios.

## Figures and Tables

**Table 1:** Participants’ driving and drinking-related characteristics.

Characteristic	Mean ± SD *	Min	Max
Age [years]	38.7 ± 12.3	26	58
Weight [kg]	67.0 ± 11.0	55	95
Height [cm]	172.6 ± 12.8	165	178
Driving experience [years]	19.4 ± 12.8	5	39
Driving distance [km/year]	6480 ± 5780	0	18,000
Frequency of alcohol consumption [1~9] ^†^	5.7 ± 1.3	5	8
Average alcohol consumption [mL]	50.0 ± 32.5	25	125

* Standard deviation. ^†^ Frequency of alcohol consumption, labeled from 1 to 9, represents a gradual decrease in frequency. Their definitions are as follows: more than 1 time every day; 1 time everyday; 5~6 times a week; 3~4 times a week; 1~2 times a week; 3 times a month; 2 times a month; 1 time a month; less than once a month.

**Table 2:** Implementation details of the selected baseline methods.

Model	Framework	Hyperparameters
LDA	scikit-learn [51]	significant threshold tol=0.0001
AdaBoost	scikit-learn [51]	max number of estimators n_estimators=100
		learning_rate=0.05
XGBoost	XGBoost [52]	max number of estimators n_estimators=100
		learning_rate=0.3
SVM	cuML [53]	regularization parameter C=0.0039
		σ=8 in kernel coefficient
RF	cuML [53]	number of trees n_estimator=200
		max depth of the tree max_depth=8
		min_samples_split=2 to split an internal node
VAE	PyTorch [50] scikit-learn [51]	symmetric encoder/decoder en_layers=(64,128,256,512,1024)
		latent distribution dimension latent_dim=2048
		learning rate of Adam lr=0.004
		step scheduler step=50, decay=0.95
		isolation forest contamination=0.01

**Table 3:** Statistical and performance metric comparison with baseline methods in terms of detecting DUI driving behavior in normal and drunk records across all three routes.

Model	Minimum	Median	Maximum	IQR	AUC ↑	*p*-Value ↓
LDA *	0.0236/0.0505	0.1741/0.1905	0.2996/0.3958	0.1083/0.1560	0.581	0.218
	(+0.0269)	(+0.0163)	(+0.0962)	(+0.0476)		
SVM *	0.0341/0.0666	0.1069/0.1921	0.2907/0.6850	0.1116/0.2639	0.762	0.001
	(+0.0325)	(+0.0851)	(+0.3943)	(+0.1522)		
RF *	0.0305/0.0688	0.1530/0.2666	0.3484/0.6975	0.1054/0.2545	0.760	**<0.001**
	(+0.0384)	(+0.1136)	(+0.3492)	(+0.1492)		
ADA *	0.0803/0.1097	0.1545/0.2867	0.3801/0.6601	0.0999/0.2328	0.753	0.002
	(+0.0295)	(+0.1322)	(+0.2800)	(+0.1329)		
XGB *	0.1054/0.1283	0.1901/0.3380	0.4669/0.7873	0.1355/0.2517	0.751	0.002
	(+0.0229)	(+0.1479)	(+0.3203)	(+0.1162)		
VAE	0.0001/0.0000	0.0065/0.0103	0.0202/0.0355	0.0088/0.0144	0.588	0.051
	(−0.0001)	(+0.0038)	(+0.0153)	(+0.0056)		
DUIncoderG	0.0029/0.0023	0.0078/0.0127	0.0198/0.0502	0.0069/0.0213	0.657	0.004
	(−0.0006)	(+0.0050)	(+0.0304)	(+0.0143)		
DUIncoderR	0.008/0.0267	0.0125/0.1519	0.1970/0.3613	0.0773/0.1234	**0.847**	**<0.001**
	(+0.0260)	(+0.1394)	(+0.1643)	(+0.0461)		

The element of the statistical values (minimum, median, maximum, IQR) in the table represent value from normal/drunk group, respectively, and the values in parentheses represent the difference between normal and drunk. ↑ larger value signifies better results. ↓ smaller value signifies better results. * Supervised classifiers which require both normal and drunk data for training.

**Table 4:** Performance metric comparison with baseline methods in terms of detecting DUI driving behavior in normal and drunk records of three different routes, respectively.

Model	Accident		Urban		Highway	
	AUC ↑	*p* -Value ↓	AUC ↑	*p*-Value ↓	AUC ↑	*p*-Value ↓
LDA *	0.656	0.214	0.589	0.403	0.600	0.454
SVM *	0.767	0.076	0.689	0.180	0.833	0.015
RF *	0.778	0.072	0.789	0.051	0.789	0.013
ADA *	0.722	0.121	0.767	0.124	0.789	0.033
XGB *	0.744	0.098	0.778	0.123	0.756	0.023
VAE	0.722	0.077	0.589	0.262	0.500	0.404
DUIncoderG	0.833	0.005	0.622	0.212	0.733	0.111
DUIncoderR	**0.889**	**0.001**	**0.878**	**0.007**	**0.867**	**0.011**

↑ larger value signifies better results. ↓ smaller value signifies better results. * Supervised classifiers which require both normal and drunk data for training.

**Table 5:** Performance metric comparison of DUI behavior detection by DUIncoders trained on different scenarios in normal and drunk data for three routes.

Model	Training			Evaluation							
	A	U	H	All		Accident		Urban		Highway	
				AUC ↑	*p*-Value ↓	AUC ↑	*p*-Value ↓	AUC ↑	*p*-Value ↓	AUC ↑	*p*-Value ↓
${DUI}_{G}$	✓			0.584	0.923	0.878	0.010	0.567	0.580	0.600	0.390
		✓		0.531	0.922	0.589	0.350	0.622	0.202	0.567	0.431
			✓	0.525	0.928	0.578	0.435	0.556	0.522	0.511	0.737
	✓	✓		0.599	0.904	0.867	0.012	0.589	0.175	0.578	0.553
	✓	✓	✓	0.677	0.002	0.833	0.006	0.622	0.212	0.733	0.112
${DUI}_{R}$	✓			0.590	0.090	0.922	<0.001	0.600	0.284	0.644	0.393
		✓		0.575	0.433	0.711	0.099	0.878	0.003	0.589	0.601
			✓	0.538	0.905	0.644	0.414	0.589	0.369	0.500	0.641
	✓	✓		0.680	0.049	0.911	0.001	0.856	0.001	0.611	0.511
	✓	✓	✓	0.851	<0.001	0.889	0.001	0.878	0.007	0.867	0.012

In the context of “Training”, “A”, “U”, and “H” represent the data types used during training, specifically “accident”, “urban”, and “highway”, respectively. ↑ larger value signifies better results. ↓ smaller value signifies better results.

**Table 6:** Performance metric comparison of DUI behavior detection by DUIncoders using different size of sliding window for route “accident” and route “urban”.

Model	Window Size	AUC ↑	*p*-Value ↓
DUIncoderG	5 s	0.772	0.002
	10 s	0.703	0.006
	15 s	0.733	0.006
	20 s	0.748	0.004
DUIncoderR	5 s	0.867	<0.001
	10 s	0.878	<0.001
	15 s	0.881	<0.001
	20 s	0.886	<0.001

↑ larger value signifies better results. ↓ smaller value signifies better results.

**Table 7:** Performance metric comparison of DUI behavior detection by DUIncoders using different hyperparameter settings of novelty detector for route “accident” and route “urban”.

Model	Contamination	AUC ↑	*p*-Value ↓
DUIncoderG	0.001	0.721	0.003
	0.005	0.711	0.005
	0.01	0.703	0.007
	0.05	0.694	0.017
	0.1	0.686	0.028
DUIncoderR	0.001	0.872	<0.001
	0.005	0.872	<0.001
	0.01	0.878	<0.001
	0.05	0.894	<0.001
	0.1	0.894	<0.001

↑ larger value signifies better results. ↓ smaller value signifies better results.

**Table 8:** Performance metric comparison of DUI behavior detection by DUIncoders using different proportions of training data for route “accident” and route “urban”.

Model	Data Proportion	AUC ↑	*p*-Value ↓
DUIncoderG	20%	0.722	0.004
	40%	0.750	0.003
	60%	0.725	0.004
	80%	0.733	0.003
	100%	0.703	0.006
DUIncoderR	20%	0.806	0.001
	40%	0.869	<0.001
	60%	0.872	<0.001
	80%	0.864	<0.001
	100%	0.878	<0.001

↑ larger value signifies better results. ↓ smaller value signifies better results.

**Table 9:** Inference time and memory consumption for DUIncoder with different devices.

CPU	GPU	Memory [MB]	Time [ms]
Intel E5-2697 v3	NVIDIA GeForce RTX 2080 Ti	327.83	1510.0
Intel E5-2697 v3	NVIDIA TITAN X (Pascal)	327.83	1791.2
Intel E5-2699 v4	NVIDIA RTX A6000	327.83	1185.4
AMD EPYC 7542	NVIDIA GeForce RTX 3090	327.83	1125.0

As the experimental platform is used by multiple researchers, the inference time may vary due to the influence of concurrent tasks.

**Figure A1:**
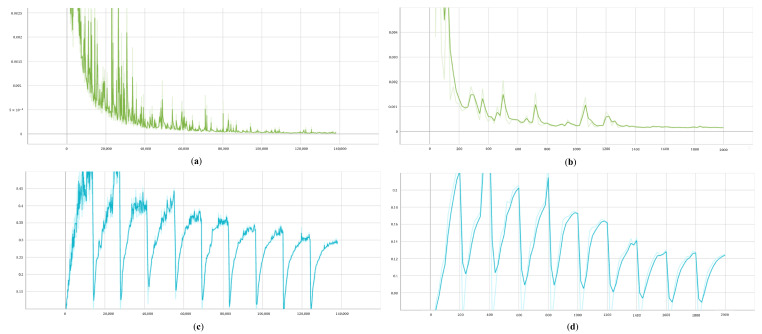
Training and validation loss curves for driver models in DUIncoders: (**a**) training loss in DUIncoderR; (**b**) validation loss in DUIncoderR; (**c**) training loss in DUIncoderG; (**d**) validation loss in DUIncoderG.

## Data Availability

The dataset utilized in this project was collected from the driving simulator in the Advanced Research and Innovation Center, DENSO CORPORATION. This dataset has been specifically provided for the purpose of conducting various experiments within this project. Code will be available at https://github.com/HanaRo/DUIncoder (accessed on 4 March 2025).

## Abbreviations

The following abbreviations are used in this manuscript:

DUI	Driving Under Influence
WHO	World Health Organization
DL	Deep Learning
DNN	Deep Neural Network
BAC	Blood Alcohol Content
CAN	Controller Area Network
LDA	Linear Discriminant Analysis
MLP	Multi-Layer Perceptron
SVM	Support Vector Machine
RF	Random Forest
CNN	Convolutional Neural Network
VAE	Variational Autoencoder
ELBO	Evidence Lower Bound
DoF	Degrees of Freedom
AnoP	Anomalous Proportion
IQR	Interquartile Range
AUC	Area Under the Curve
ROC	Receiver Operating Characteristic
LLM	Large Language Model
VLM	Vision-Language Model

## Appendix A. Training and Validation Losses

Section 3.3.3 provides the implementation process of DUIncoder, which includes two distinct procedures for training and validating the driver model. To enhance comprehension of the training and validation procedures of the driver model in DUIncoders, Figure A1 illustrates the loss curves for both DUIncoderG and DUIncoderR. The *y*-axis in all subfigures denotes the loss function. The *x*-axis in Figure A1a,c is measured in steps, whereas in Figure A1b,d, it is measured in epochs.

As stated in Section 5.1.1, DUIncoderR only employs the reconstruction error $L_{\mathrm{recon}}$ as its loss function, which gradually decreases throughout training. In contrast, the loss function of DUIncoderG exhibits periodic characteristics due to the cyclic variations in the ELBO loss $L_{\mathrm{gauss}}$, which comes from a cosine annealer [49]. However, it also follows an overall downward trend. Compared to training, the driver model performs multiple sampling operations during validation to compute the frame-wise minimal deviation. DUIncoderR maintains a low error below 0.0005 in both training and validation. DUIncoderG, benefiting from its better generative diversity, achieves a lower error in validation (below 0.08) compared to training (below 0.15). As mentioned in Section 3.3.3 and Section 5.1.1, we divided the entire DUI dataset into three subsets and selected the best performing model on the validation set to prevent overfitting. In the experiment, DUIncoderG achieved its optimal performance at epoch 1860 with a validation loss of 0.00016; DUIncoderR reached its best performance at epoch 1820 with a validation loss of 0.03754.
